# Disentangling the role of environment in cross-taxon congruence of species richness along elevational gradients

**DOI:** 10.1038/s41598-021-83763-3

**Published:** 2021-02-25

**Authors:** Carolina S. Ramos, Pablo Picca, Martina E. Pocco, Julieta Filloy

**Affiliations:** 1 Departamento de Ecología, Genética y Evolución, Facultad de Ciencias Exactas y Naturales, Universidad de Buenos Aires, Buenos Aires, Argentina; 2 Departamento de Biodiversidad y Biología Experimental, Facultad de Ciencias Exactas y Naturales, Universidad de Buenos Aires, Buenos Aires, Argentina; 3 Centro de Estudios Parasitológicos y de Vectores (CEPAVE), CONICET-UNLP, La Plata, Argentina; 4 División Entomología, Museo de La Plata, Universidad Nacional de La Plata, La Plata, Argentina; 5 Instituto de Ecología, Genetica y Evolución (IEGEBA), Consejo Nacional de Investigaciones Científicas y Técnicas (CONICET) - Universidad de Buenos Aires, Buenos Aires, Argentina; 6 Instituto de Micología y Botánica (INMIBO), Consejo Nacional de Investigaciones Científicas y Técnicas (CONICET, Universidad de Buenos Aires, Buenos Aires, Argentina

**Keywords:** Biodiversity, Biogeography, Community ecology, Macroecology, Theoretical ecology

## Abstract

Spatial patterns of species richness have been found to be positively associated, a phenom called cross-taxon congruence. This may be explained by a common response to environment or by ecological interactions between taxa. Spatial changes in species richness are related to energy and environmental heterogeneity but their roles in cross-taxon congruence remain poorly explored. Elevational gradients provide a great opportunity to shed light on the underlying drivers of species richness patterns. We study the joint influence of environment and biotic interactions in shaping the cross-taxon congruence of plants and orthopterans species richness, along three elevational gradients in Sierras Grandes, central Argentina. Elevational patterns of species richness of orthopterans and plants were congruent, being temperature the best single predictor of both patterns supporting the energy-related hypotheses. Using a structural equation model, we found that temperature explained plant richness directly and orthopteran richness indirectly via orthopteran abundance. Cross-taxon congruence is likely due to a common response of both taxa to temperature although via different theoretical mechanisms, possibly, range limitations for plants and foraging activity for orthopterans. We disentangled the role of temperature in determining the cross-taxon congruence of plants and orthopterans by showing that a common response to the environment may mask different mechanisms driving the diversity of different taxonomic groups.

## Introduction

Understanding the main drivers of geographic patterns in species richness represents one of the most significant challenges facing ecologists and biogeographers^1–3^. It is known that species richness of different taxa are often correlated, a phenomenon called cross-taxon congruence^4^. It was proposed that cross-taxon congruence can be given by a common response to environmental factors or by an effect of the diversity of one taxon on the diversity of another due to trophic interactions or functional interdependence^4,5^. Particularly, disentangling factors explaining species diversity and the degree of congruence between them would provide a wide framework to understand the main drivers of diversity patterns^6^. Furthermore, the study of cross-taxon congruence has been proposed as a key issue in conservation ecology since the effective use of indicator taxa depends on those patterns^7^. Thus, understanding the role of environment in the cross-taxon congruence may also contribute to evaluating the effectiveness of those indicator taxa.

Elevational gradients provide a great opportunity to shed light on the underlying drivers of species richness patterns^8–10^. For instance, they exhibit large environmental variation over a short geographic distance, offer the possibility to find replicates^6^, to select a specific environmental scenario, and to avoid covariation between alternative explicative factors^9^. Besides, changes in seasonality that occur in latitudinal gradients can be avoided along elevation gradients^11^. Moreover, mountains host an over proportional fraction of global biodiversity^3,6,12^ and are particularly vulnerable to climate change^13^. Elevational gradients were the focus of many studies and the altitudinal richness patterns they described were quite variable, but McCain^9^ proposed to summarize them in four classes: decreasing diversity with increasing elevation (decreasing), high diversity across a plateau of lower elevations then decreasing monotonically (low elevational plateau), increasing diversity at lower elevations and then decreasing (low elevational plateau with mid-peak), a unimodal pattern with maximum diversity at intermediate elevations (unimodal mid elevational peak). Here, we analyzed the degree of cross-taxon congruence between elevational patterns of species richness in vascular plants and orthopterans and whether it resembles with one of the four classes of patterns described.

The environmental factors potentially driving species richness patterns, and therefore key to understanding the cross-taxon congruence, can be grouped in energy-related hypotheses^1,4,14,15^ or habitat heterogeneity-related hypotheses^16,17^. Energy-related hypotheses have the strongest support to explain spatial diversity patterns but the mechanisms underlying those patterns are still elusive^18^. These hypotheses propose that energy limits species richness through at least three different mechanisms. First, the ambient energy hypothesis, states that temperature limits species richness through physiological constraints associated with species physiological tolerance^14,15^. Hence, temperature is expected to show a positive and direct effect on species richness. Second, the species-productivity hypothesis^14^ proposes that energy flowing through food webs is limiting richness; water-energy dynamics limit plant richness and herbivore richness is limited by the net primary production of plants and so on up the food chain. Species richness increases because an increase in resources may promote large population sizes and then the probability of local extinctions is reduced^19^. Therefore, we expect a positive and indirect effect of net primary production on species richness mediated by species abundance. Third, the temperature-mediated resource exploitation hypothesis states that temperature can be limiting the exploitation of the available resources^2,20^. This limitation reduces species abundance and therefore species richness. Alternatively, the species-heterogeneity hypothesis found wide support to explain spatial patterns in species richness^16^. This hypothesis states that an increase in the number of habitat types or structural complexity should increase the available niche space and thus allow more species to coexist^16,21^. Particularly in mountain systems, a great slope is related to a major micro-environmental gradient and major microhabitat complexity^22^. Thus, if this hypothesis prevailed, a positive relationship between species richness and habitat heterogeneity measures would be expected, irrespective of the altitudinal level where maximum values of those measures occur in each elevational gradient.

Here, we simultaneously explored the described hypothesis to disentangle the processes underlying the relationship between the environment and the cross-taxon congruence. We particularly asked (1) What is the strength of the correlation between the altitudinal richness patterns of plants and orthopterans? (2) What is the shape of these altitudinal patterns? (3) Which of the species richness hypotheses proposed finds greatest support? (4) The congruence pattern is an effect of the diversity of one taxon on the diversity of another taxon or a response of both taxa to the same environmental factor? We used a structural equation model (SEM) approach to contrast support of the alternative hypotheses proposed to explain the cross-taxon congruence. Plants are the basis of trophic cascades and provide a great variety of resources for herbivores, such as orthopterans, which provided us with an excellent model to analyze the existence of direct relationships of one taxon over another as a possible cause of the congruence of richness patterns. Moreover, we selected elevational gradients where temperature, primary productivity, and environmental heterogeneity were not correlated to reveal their roles. Overall, this study builds toward a better understanding of elevational richness patterns and the joint influence of biotic and abiotic factors shaping such patterns.

## Results

### Elevational patterns of environmental variables

**Figure 1:**
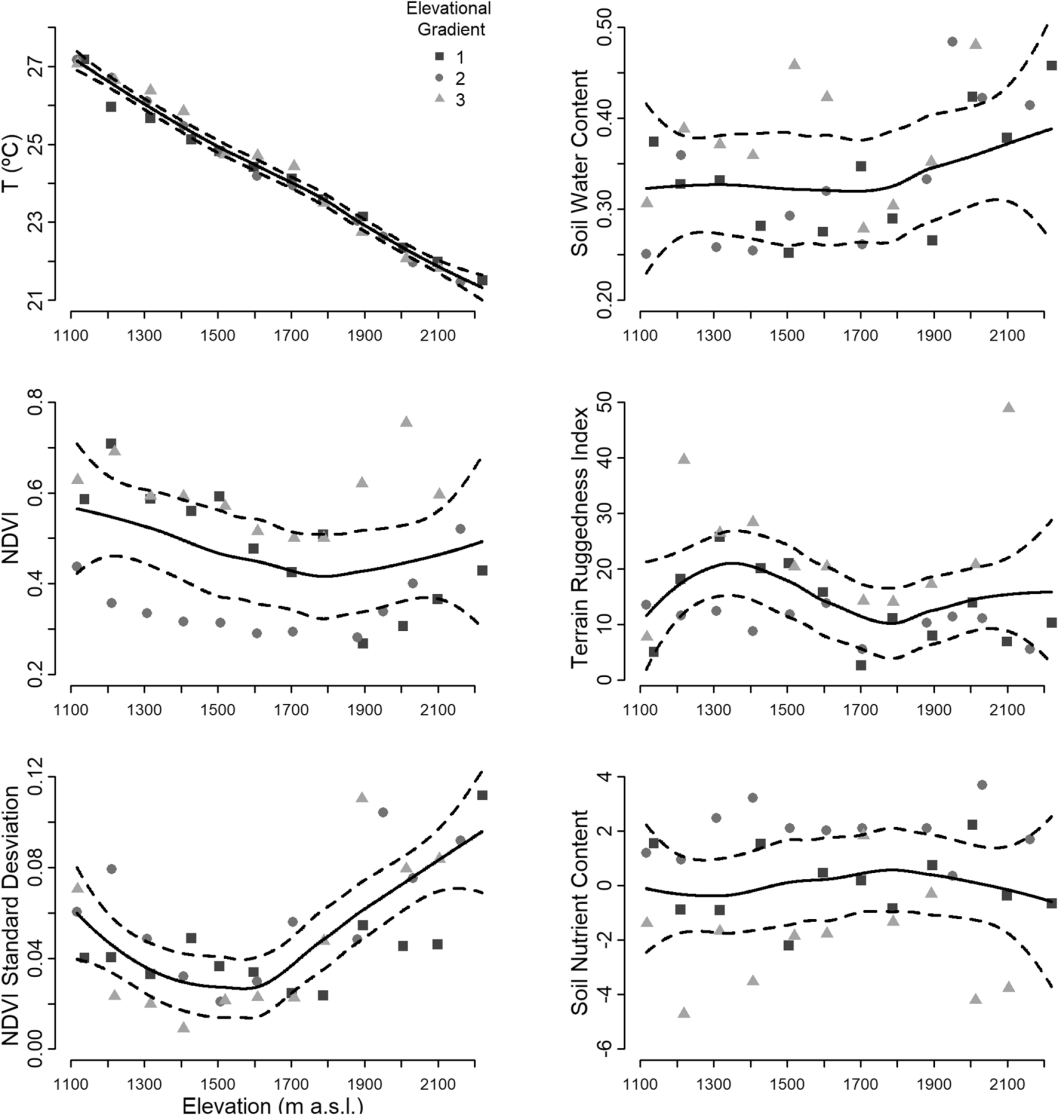
Elevational patterns of each environmental variable for the three gradients. The line is a local regression model for the three mountains together with a 95% confidence interval. Figure generated using R software^23^.

Different elevational patterns were observed for the environmental variables considered (Fig. 1). Temperature decreased as the elevation increased. Soil moisture showed a relatively constant pattern until it reached 1800 m a.s.l. where it began to increase. NDVI decreased to 1800 m a.s.l. and then increased, jointly with the increase in soil moisture. Terrain ruggedness index (TRI) showed a complex pattern with the lowest values at the base and at 1800 m a.s.l., peaking at 1300 m a.s.l. At high altitude TRI was variable, showing high values of ruggedness for one gradient and low values for two gradients that exhibit plateaus at the top of the mountains. The standard deviation of the NDVI showed a minimum at intermediate elevations. Finally, the soil nutrient component remained relatively constant throughout the elevational gradients.

### Species richness models and cross-taxon congruence

**Figure 2:**
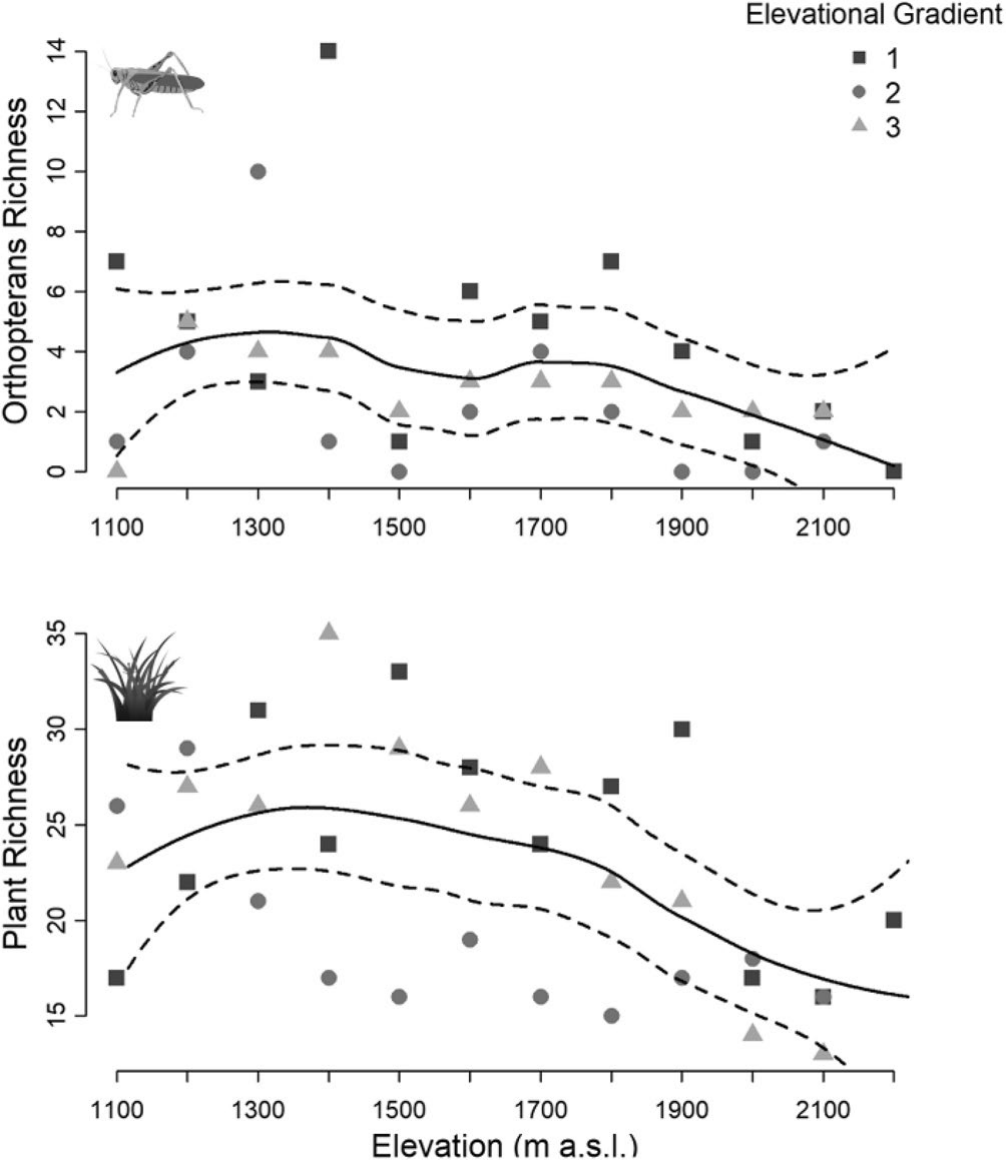
Elevational pattern of orthoptera and plant richness for each of the elevational gradients. The line is a local regression model for the three elevational gradients together with a 95% confidence interval. Spearman correlation: r = 0.37, p = 0.03. Figure generated using R software^23^.

We identified a total of 192 plant species (Supp_1) and 38 orthopteran species (Supp_2). Cross-taxon congruence was detected as the Spearman correlation in the elevational patterns of species richness was significant (r = 0.37, p = 0.03). Furthermore, for both taxa, the trend fitted by locally weighted regressions resembles to a low elevational plateau with a mid-peak pattern (Fig. 2). Across low elevations richness increased, a maximum of richness was found around the 1300 m a.s.l., and then richness decreased along the elevational gradients.

**Figure 3:**
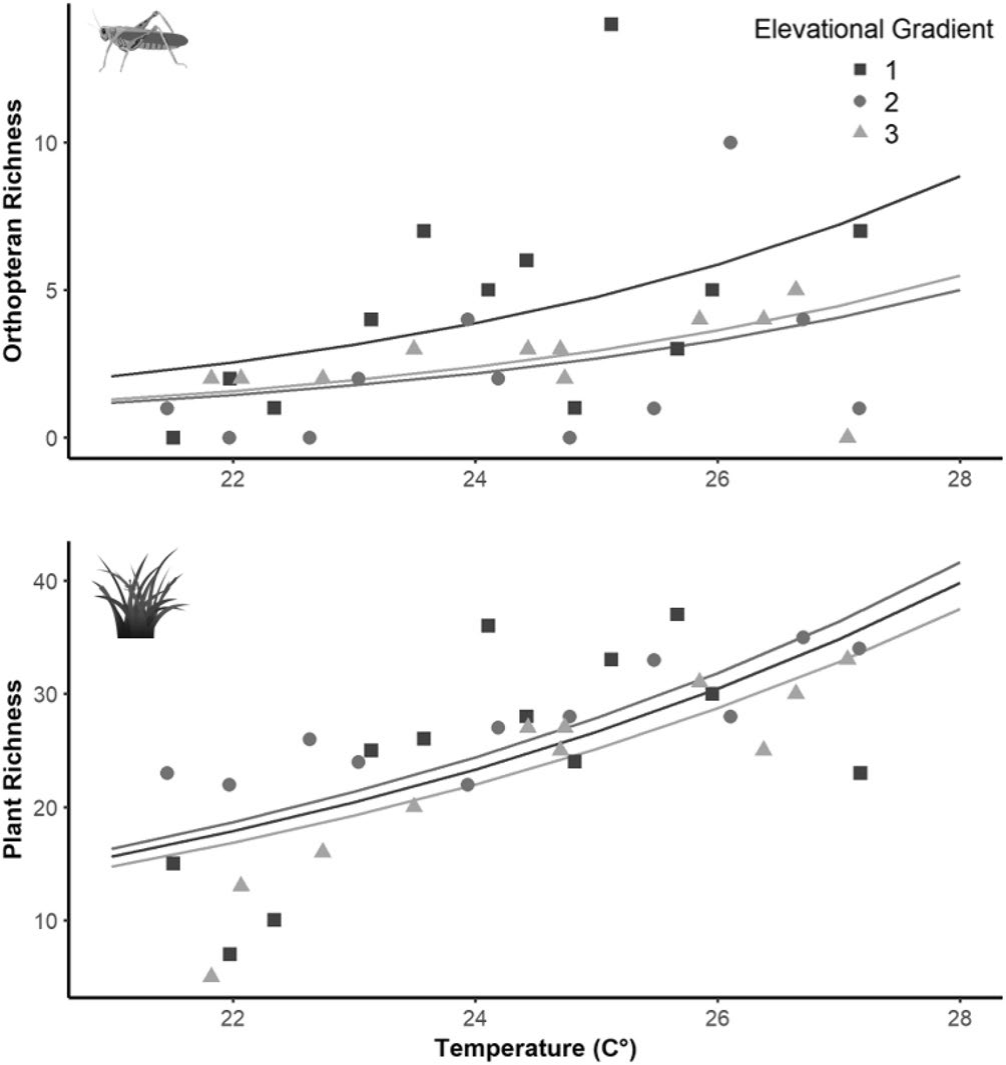
Conditional predictions for the best fitted models for orthoptera and plant richness. Figure generated using R software^23^.

The selection procedure performed to compare the degree of support of the hypotheses proposed and to identify the main single environmental variables related to elevational patterns in species richness showed that both orthopteran and plant species richness were best explained by temperature (ΔAICc < 2) (Table 1). The best fitted models showed that orthopteran and plant richness increased with increasing temperature (Fig. 3). However, the relationships between temperature and species richness appeared to be humpbacked.

**Table 1:** Model selection results of orthopteran and plant species richness.

Taxon	Model	AICc	Δ AICc	AICc w
Orthopterans	Temperature	163.495	0.000	0.917
	NDVI standard deviation	168.679	5.184	0.069
	Plant richness	173.454	9.959	0.006
	Null	174.591	11.096	0.004
	NDVI	175.223	11.728	0.003
	Terrain ruggedness	175.775	12.280	0.002
Plants	Temperature	210.939	0.000	0.986
	Soil nutrient content	220.996	10.057	0.006
	Null	221.849	10.910	0.004
	Terrain ruggedness	223.030	12.092	0.002
	Soil water content	224.130	13.191	0.001

*Coeff.* Coefficient, *AICc* Akaike information criterion corrected for small sample size, *AICc w* weights of the Akaike information criterion corrected for small sample size.

**Figure 4:**
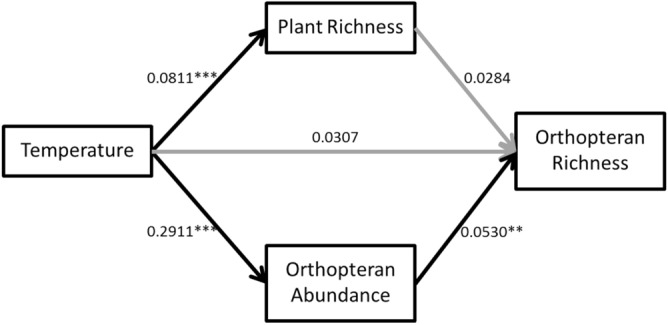
Structural equation model for the alternative mechanisms of cross-taxon congruence between orthopteran and plant richness. We show standardized partial regression coefficients and their significance levels (**p > 0.01; ***p > 0.001) shown above the arrows. Black arrows represent significant paths.

The overall evaluation of the structural equation model indicated that there were no missing paths in the model (Fisher’s C = 1.96, p-value = 0.38). Temperature showed a positive direct association with plant richness and orthopteran abundance and a positive indirect association with orthopteran richness via orthopteran abundance. But temperature is not directly related to orthopteran species richness (Fig. 4). Furthermore, plant richness is not related to orthopteran richness.

## Discussion

Richness patterns of plants and orthopterans were congruent along elevational gradients, showing both taxa a response to temperature over a potential interaction between them. However, by deepening the insight on that common response to temperature, different hypotheses arose to explain species richness of both taxa. While plant richness was directly related to temperature, supporting the ambient energy hypothesis, orthopteran richness was indirectly related to temperature via orthopterans abundance, supporting the resource-exploitation hypothesis. Our analytical framework suggested that a direct relationship between the diversity of both taxa, as a result of trophic interactions or functional interdependence, may be discarded as the main driver of cross-taxon congruence. Thus, as recently claimed, species coexistence patterns do not necessarily provide evidence of ecological interactions^24^. Moreover, the response of both taxa to a single environmental variable (i.e., temperature) via different mechanisms for both plants and orthopterans could imply that the congruence would be decoupled in a context of rapid climate change.

The elevational species richness patterns of orthopterans and plants along the Sierras Grandes could be described as a low elevational plateau with a mid-peak (i.e., increasing diversity at low elevation, peaking at low elevations, and then decreasing towards high elevation), which is an uncommon pattern for both taxa. If the shape described here reflects a possible elevational trend for othopterans, to our knowledge, this is the first time that a low elevational plateau with a mid-peak pattern was documented. Previous studies showed either a lack of response of orthopteran richness to elevational gradients^25,26^, a mid-elevational peak^27,28^ or a decreasing pattern^29,30^. For plants, although it was previously found^31^, McCain and Grytnes^32^ proposed the mid elevational peak as the most frequent pattern. At low elevation, Sierras Grandes registered a woodland belt with high levels of water, productivity, temperature and habitat complexity. Thus, those factors may be acting synergically likely driving higher diversity than typically expected for low elevations. At low-mid elevation, the ecotone between woodland and shrubland belts at approximately 1300 m a.s.l was possibly causing a peak in species richness in mountain systems due to the overlap of two communities (from low and high altitudes)^8,32^. Furthermore, the high levels of species coexistence may be maintained by topographic heterogeneity which also reached a peak in low-mid elevations. The decreasing species richness pattern, towards high elevation, is usually attributed to the decreasing of temperature and productivity with altitude^18^. In Sierras Grandes, the decreasing richness pattern above 1300 m.a.s.l. may be explained by decreasing temperature as productivity does not decrease with altitude. Particular environmental scenarios related to the history of the mountain system might be determinant for the shape of the elevational pattern of species diversity. Despite all particularities, relying on environmental conditions provides a strong framework to understand the underlying causes of those elevational patterns.

Overall, the common elevational trend in decreasing species richness for plants and orthopterans was mainly, but not exclusively as discussed in the previous paragraph, explained by decreasing temperature with elevation. Thus, our results supported temperature mediated hypotheses over productivity and environmental heterogeneity hypotheses; as previously discussed, those environmental factors may also be involved in shaping the elevational pattern (e.g.^33^). Plant richness positive response to temperature over other factors was previously found (e.g.^10^). For insects, species richness positive response to temperature was also well documented^25^. Thus, in our study system, the role of alternative factors may emerge in the lowlands where temperature is higher than in the highlands. Towards high elevation, temperature mediated processes likely obscured positive productivity-diversity and heterogeneity-diversity relationships which may be revealed where temperature is not limiting (e.g.^34^). Thus, the cross-taxon congruence reflected a response of both taxa to an environmental variable providing evidence that the correlation of spatial diversity patterns is not an indicator of functional relationships between them, as recently proposed^24^.

The cross-taxon congruence found here seemed to be the consequence of changes in both, plants and orthopterans species richness mainly related with changes in temperature. However, our SEM results showed that temperature was directly associated with plant richness and indirectly associated with orthopteran richness via orthopteran abundance. Thus, rather than a direct relationship between them; the response likely involved different underlying mechanisms driving the elevational patterns in species richness. Temperature was possible limiting plant richness via physiological tolerances at high altitude, as high-altitude belts are dominated by Andean plants likely tolerant to lower temperatures than subtropical plants occurring in lowlands. The indirect association between orthopteran richness and temperature via orthopteran abundance suggested that orthopteran activity is controlled by temperature and consequently controls their access to food. As stated in the resource exploitation hypothesis, for ectotherms, activity is limited by temperature, so foraging is greater in warm than in cold temperatures^2^. Thus, the higher the temperature, the greater the possibility of exploiting the available resources likely enhancing species abundance and richness, as proposed for bees^2^ and other arthropods^35^. Besides disentangling the potential drivers of elevational richness patterns of plants and orthopterans, we identified a causal model showing that the cross-taxon congruence is likely due to a common response of both orthopterans and plants to temperature. However, that common response to temperature involves physiological constraints with different underlying mechanisms: thermal tolerance and survivorship for plants and foraging activity for orthopterans. Therefore, temperature acted as a surrogate of environmental energy which implies range limitations mechanisms^36^ for plants and as a surrogate of the kinetic energy that provides temperature to increase activity^20^ for orthopterans. This highlights that understanding causes behind cross-taxon congruence could provide deep insights into the interplay between interspecific relationships and environmental conditions as drivers of species richness.

The cross-taxon congruence found here indicated that plants and orthopterans can be used as a surrogate of the diversity of each other, and potentially as a surrogate of biodiversity in general^5^. While covariance in species diversity provides a useful tool in conservation biology by the identification of bioindicators, our results indicated that the knowledge of the distribution patterns of one taxonomic group may not necessarily provide insight into the processes structuring the distribution patterns of other taxonomic groups. Moreover, as previously claimed, the strength of cross-taxon congruence is not consistent among many perspectives. For example, previous studies showed a stronger influence of plant richness than environmental variables on bird richness^37,38^. Qian and Kissling^41^ found that mammal and bird richness were strongly influenced by plant richness than amphibian and reptile richness and proposed that the direct relationship with plants is stronger for endothermic than ectothermic organisms. In fact, ectothermic organisms tend to be more affected by environmental conditions than endothermic organisms^39^. Also, the strength of the cross-taxon congruence between arthropods and plants seems to be influenced by the degree of specialism of the taxa involved; specialist insects (e.g., endophagous and galling) showed a greater dependence on plant richness than less specialized ones (e.g., exophagous and non-galling)^40^. The cross-taxon congruence showed to be scale dependent^5^, being stronger at large than at local scale^41^. Moreover, the relative influence of different factors in determining species richness patterns also changes with scale^41^ and also changes the relative contribution of large and small scale processes across taxa^42^, being variables related to energy those that play a preponderant role at large scale or probably at large climatic gradients^43^, like those found in mountains. For example, a moderate congruence could involve a different scale of response for both taxa to the environment. Thus, from a theoretical perspective, future investigations should focus on disentangling the drivers as well as the scale dependence of the cross-taxon congruence involving different types of organisms regarding body temperature regulation and degree of specialization. From a pragmatic perspective, irrespective of the scale and the determinant factor, plants were proposed as good indicators of species richness of other groups^7,42^ and that statement seems to hold, at least, in Sierras Grandes.

## Materials and methods

### Study area

This study was conducted on the western slope of the Sierras Grandes mountain range, in central Argentina. These mountains extend from north to south and their elevation varies from ca. 900–2790 m a.s.l. with an average maximum elevation of approximately 2000 m a.s.l. The climate in the study area is mountainous sub-humid to semiarid^44^. The mean annual temperature is 14 °C, decreasing to 8 °C at about 2000 m a.s.l, with no frost-free period^45^ being the rainy period from September to March^46^.

The area corresponds to the Mountain Chaco district of the Chaco biogeographic region^47^ and historically three different elevational belts were described, (1) mountain woodland, reaches up to 1300 m a.s.l., (2) mountain shrublands, distributed between 1300 and 1700^44^, and (3) high mountain grassland above 1700^45^. The mountain woodland is dominated by tree species such as *Lithraea molleoides, Acacia caven*, and *Fagara coco*^48^*.* The mountain shrubland is dominated by *Baccharis aliena*^46^*.* The high mountain grassland up to 1900 m a.s.l. is dominated mainly by *Stipa* spp.*, Piptochaetium* spp. and *Festuca hieronymi.* Above 1900 m a.s.l. the predominant species are *Deyeuxia hieronymi*, *Poa stuckertii* and *Alchemilla pinnata*^45^*.* Considering species historical biogeography, grasslands at lower altitude are dominated by subtropical grass genera whereas Andean taxa predominate above 1900 m a.s.l^45^. Recently, due to the difference in the evolutionary origins of the species composing the assemblages at high and low altitudes, this area was proposed as a part of a new biogeographic province namely Comechingones^49^. Livestock rearing is the main economic activity since the seventeenth century in Sierras Grandes however, they are in a relatively good state of conservation^44,46^.

### Study design

**Figure 5:**
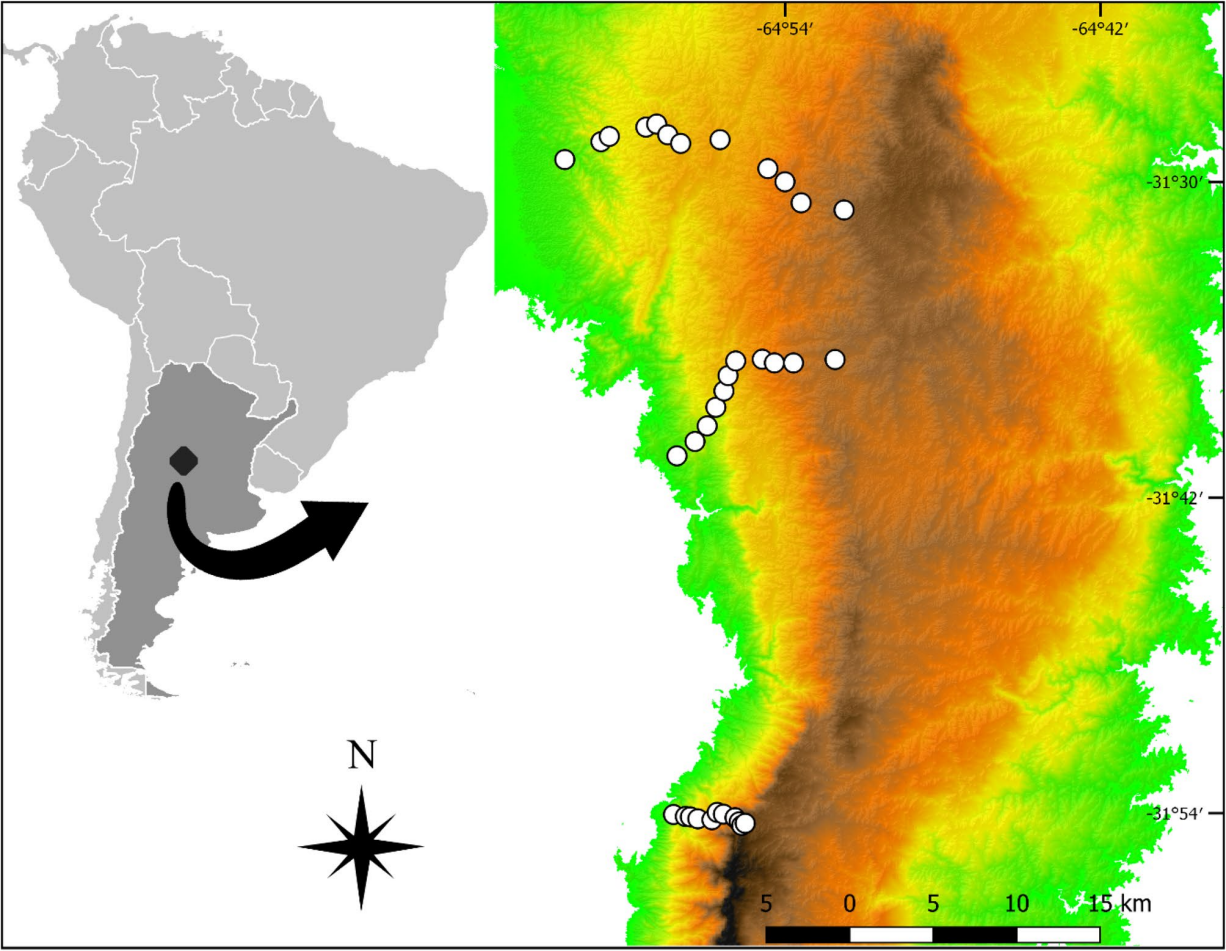
Location of the study sites in Sierras Grandes Mountains in central Argentina. Map created using the Free and Open Source QGIS^50^.

We identified all the pre-existing ascent trails of the area by using satellite images and tourist information and those mostly covering the elevation range were selected. We kept three elevational trails (elevational gradients), ordered from north to south: (1) Cerro Los Gigantes, spanning from 1100 to 2200 m a.s.l., (2) Cerro Trinidad, 1100 to 2100 m a.s.l. and (3) Cerro Ventana, 1100 a 2100 m a.s.l. We located a sampling site every 100 m a.s.l. along each gradient (Fig. 5). Each sampling site consisted of an observational unit where orthopterans and plants were sampled and environmental variables were measured.

### Orthoptera and plant sampling

Based on Kent^51^ plant species were surveyed using the Braun–Blanquet method at each sampling site in three quadrats (1 m × 1 m for herbs and of 4 m × 4 m for woody plants) in well-defined homogeneous vegetation strata, avoiding non-representative small vegetation patches. Specimens were collected for further identification and some species were taxonomically identified in the field. Plant taxonomy follows the Catalogo de Plantas Vasculares del Cono Sur^52^ and the online update (http://www.darwin.edu.ar). Orthopterans were collected with an entomological net (diameter: 38 cm) on three occasions during summer^28^ by applying 50 sweeps along a 50 m line at each sampling site. This widely used technique allows a representative sampling of the arthropods found on the vegetation^53^. All specimens, adults and nymphs, were identified to species level when possible, using morphological characteristics according to current taxonomy^54–56^. Voucher specimens of all morphospecies have been deposited at Museo de La Plata (MLP), Argentina.

### Estimation of environmental variables

We considered three variables related to the energy hypotheses and two variables related to the habitat heterogeneity hypothesis. As energy-related variables, we estimated temperature, water availability, and net primary productivity (NPP). Mean summer temperature data was derived from WorldClim V.II^57^ with a spatial resolution of 30 s (~ 1 km^2^ cell grid)^57^, an improved version of WorldClim database^58^, widely used in mountains^12,59^. This original WorldClim database used data of climatic stations and altitude to develop interpolated climate surfaces^58^, the new version includes new variables in the interpolation process, coast distance, and three satellite‐derived covariates: maximum and minimum land surface temperature and cloud cover, obtained with the MODIS satellite platform^57^. Temperature data was resampled regarding altitude to obtain a grid resolution of 30 × 30 m^2^. Thus, we calculated the mean summer temperature in windows of 2 × 2 pixels containing each sampling site. To represent water availability for plants along the gradient in the sampling period, we took 2 soil samples in random points at each sampling site once a month during summer, to represent conditions during the growth period for plants in the study area. We estimated the mass of water per mass of dry soil using a sample taken from the top 10 cm, a widely used standard for asses soil conditions^60^. We weighting and drying the sample to remove water and then weighing the dried soil^61^. As a surrogate of the NPP, we used the normalized difference vegetation index (NDVI)^62^, which was estimated from Landsat 8 (OLI) satellite image data from the summer of the sampling year and the previous one. The spatial resolution of Landsat 8 (OLI) is 30 × 30 m^2^. Thus, the mean value of the NDVI was calculated in a window of 2 × 2 pixels, as using a group of pixels enhances the correlation of NDVI with NPP compared with pixel-level estimations^63^. Finally, to assess environmental heterogeneity we obtained the standard deviation of the NDVI to account for vegetation heterogeneity^64^. Also, environmental heterogeneity in mountain systems can be quantified as topographic heterogeneity^12,16^, an indicator of the diversity in environmental conditions over distance**.** Therefore, we first quantified the slope from a digital elevation model (DEM) of 30 × 30 m^2^ of resolution^65^ and then we calculated the terrain ruggedness index (TRI)^66^ with the spatial resolution of the DEM. TRI is related to the slope of a site and its variability^66^. At the same time, the slope is related to the elevation range of a site; a high slope implies a high elevation range and high environmental heterogeneity^67^. Thus, to estimate TRI at each sampling site, a mean TRI in a window of 2 × 2 pixels was obtained. All grid variables were calculated using QGis software^50^.

At the local scale, plant diversity may be influenced by soil characteristics^68^. Thus, to control for that factor, a sample soil was collected from the top 10 cm at each of the three plant sampling quadrats, during the survey of plants. Then, we mixed the three samples from each sampling site. The samples were analyzed in the “Instituto del Suelo” of the “Instituto Nacional de Tecnologia Agropecuaria”. The pH and electrical conductivity were determined in a 1:2.5 (w:v) suspension of soil in water. Carbon content (C) was determined by the Walkley–Black method and the conversion to organic carbon was made by a factor of 1.22. Organic nitrogen (N) (%) was ascertained by the micro-Kjeldahl procedure and total phosphorus (P) (%) by the Bray–Kurtz method. To summarize soil characteristics into a single nutrient variable, accounting for high positive correlations among all nutrient-related variables, we performed a principal component analysis (PCA) and retained the first component as a new synthetic nutrient content variable.

### Data analysis

Variables trends along the three elevational gradients were described by elaborating a scatter plot for each environmental variable as a function of elevation. Locally weighted regressions were fitted and trends were overlapped on the plots to visually analyze the elevational pattern of each environmental variable^69^. The locally weighted regressions were fitted with a span of 0.75, a second-order polynomial degree and, were fitted by least squares with *loess* function using R software^23^.

Also, we assessed the cross-taxon congruence and described the elevational trends in species richness. For orthopterans, species richness and abundance per sampling site were calculated by pooling the three summer samplings. For plants, species richness was obtained as the total number of the surveyed species by pooling the three herb plant quadrats and the three woody plants quadrats per sampling site. Then, the overall degree of cross-taxon congruence between plants and orthopterans richness was assessed by obtaining the Spearman correlation coefficient^5^. To describe the elevational trend of species richness we proceeded as previously detailed for environmental variables. We plotted species richness as a function of elevation for each taxon and fitted the trend with locally weighted regressions. For plants and orthopterans we assigned visually the obtained patterns^22,70^ to one of the four categories proposed by McCain^9^: (1) decreasing, (2) low elevational plateau, (3) low elevational plateau with a mid-peak and (4) unimodal mid elevational peak.

We analyzed the degree of support for each hypothesis by identifying, through a model selection procedure, the main environmental variable related to species richness. We ran single Generalized Linear Mixed Models (GLMM) of species richness as a function of each environmental variable,^71^ with a Poisson error distribution and included the three gradients as a random effect of a categorical factor^72^. For orthopterans, we ran six single regression models (Table 2) and one null model with no variables, to account for the lack of pattern. For plant species richness, we ran four single models (Table 2) and one null model. We ranked the models for each taxon following the Akaike information criterion using the model.sel function of the R package MuMin and kept the variable involved in the most informative one (i.e., lowest AIC) for the following analyses. Previously, environmental variables were tested for multicollinearity, using pairwise correlations and checking that the correlation coefficient was less than 0.75, to avoid spurious model selection, and to ensure that the environmental scenario was appropriated to disentangle the response of richness to the different environmental variables.

**Table 2:** Alternative environmental variables considered in the study to explain elevational species richness patterns.

Variables	Orthopterans	Plants
Temperature	X	X
Soil water content	–	X
NDVI	X	–
NDVI standard deviation	X	–
Terrain ruggedness index	X	X
Plant richness	X	–
Soil nutrient content	–	X

“X”: Variable included in the models; “–”: Variable not included in the models for not representing a hypothesis for the specified taxa.

Finally, to disentangle the role of environmental variables and biotic interactions in the cross-taxon congruence, we used structural equation modeling (SEM). We built a SEM including all possible paths linking the previously identified environmental variable with species richness for both taxa according to the theoretical hypotheses studied. This technique, contrary to a multiple regression approach, allowed the inclusion of paths representing direct and indirect relationships between variables in the model^73^. We adopted the strictly confirmatory model evaluation type^73^. Thus, we analyzed a unique complete model including paths reflecting the direct and indirect relationships among variables under the action of the ecological mechanisms driving species richness proposed by the study hypotheses. Particularly, the model included a direct relationship between plant and orthopteran species richness and a common response of both taxa to an environmental gradient to simultaneously represent the possible causes of cross-taxon congruence. We included the main environmental variables related to species richness obtained from the GLMMs for each taxon. Also, the complete SEM model included the indirect influence of environment on orthopteran species richness via orthopteran abundance to account for the resource exploitation hypothesis prediction. As we run a unique complete model we first evaluated the goodness of fit of the overall model using Shipley’s test of directed separation, which yields a Fisher’s C statistic and a Chi-square distribution^74^. Finally, if the model is adequate, the analysis should focus on estimating the strengths of the direct and indirect pathways rather than alternative partial models^73,75^. Non-significant paths were taken as not relevant relationships^73^ and would lead to discarding the hypotheses they reflect. We included the gradient as a random term and the Poisson error distribution in fitting the SEM with the PIECEWISESEM package^73^ in R software^23^.

All analyses were undertaken in the R statistical language version 3.6.2^23^. Details about the packages and functions used are given under each section. We have made our code publicly available on GitHub: https://github.com/Carolinasamanta/RichnessPattern.

## Supplementary Information

Supplementary Information 1.

Supplementary Information 2.
